# Habitat Characteristics, Distribution, and Abundance of *Cicindelidia haemorrhagica* (LeConte) (Coleoptera: Cicindelidae) in Yellowstone National Park

**DOI:** 10.3390/insects15010015

**Published:** 2023-12-29

**Authors:** Kelly A. Willemssens, John L. Bowley, Laissa Cavallini, Erik Oberg, Robert K. D. Peterson, Leon G. Higley

**Affiliations:** 1 School of Natural Resources, University of Nebraska, Lincoln, NE 68198, USA; kelly_willemssens@hotmail.com; 2 Department of Land Resources & Environmental Sciences, Montana State University, Bozeman, MT 59717, USA; johnlawrencebowley@gmail.com (J.L.B.); laissa.c@hotmail.com (L.C.); bpeterson@montana.edu (R.K.D.P.); 3 National Park Service, Yellowstone National Park, WY 82190, USA; erikoberg5@gmail.com

**Keywords:** tiger beetle, extremophile

## Abstract

**Simple Summary:**

The tiger beetle, *Cicindelidia haemorrhagica* (LeConte), occurs in the western USA and is associated with wet salt lakes. However, in Yellowstone National Park (YNP) it was observed in a thermal pool in 1891. We examined multiple thermal and non-thermal areas in YNP and discovered that *C. haemorrhagica* was exclusively associated with thermal springs, both alkaline (as high as pH 9.5) and acid (as low as pH 2.7) and at temperatures as high as 70 °C. Ultimately, we identified 17 locations with *C. haemorrhagica* populations. We estimated population sizes at multiple springs as greater than 500 individuals at each site. Additionally, we noted that beetles occurred both in the (hot) water of thermal springs and within 8 m of the spring.

**Abstract:**

We observed the tiger beetle species, *Cicindelidia haemorrhagica* (LeConte), foraging in and reproducing near the thermal pools of Yellowstone National Park (YNP). Although this species was recorded in YNP more than 130 years ago, its distribution, ecology, and association with thermal features are unknown. Therefore, we examined the distribution and habitat characteristics of *C. haemorrhagica* and evaluated methods for studying its abundance. Given the extreme environments in which these beetles live, typical methods to estimate abundance are challenging. We used a series of presence/absence studies and observations to assess distribution and recorded temperature and pH measurements to determine habitat characteristics. We also conducted visual counts, light trapping, and mark/recapture experiments to assess abundance. The inability to capture *C. haemorrhagica* with lights led to a phototaxis experiment, which showed minimal attraction to light. *Cicindelidia haemorrhagica* was found throughout YNP, but it was exclusively associated with thermal springs. The thermal springs ranged from pH 2.7 to 9.0 with temperatures from 29.1 to 75.0 °C and had varying metal concentrations in soil and water. However, all thermal springs with *C. haemorrhagica* had barren soil with a gradual slope toward the thermal water. Specifically, habitats were thermal pools with gradual margins (a less than five-degree slope) and thermal (i.e., heated) soils for larval burrows by thermal springs or pools. Population sizes of *C. haemorrhagica* ranged between 500 and 1500 individuals based on visual counts.

## 1. Introduction

Understanding the biology of organisms associated with thermal features in Yellowstone National Park (YNP) has been of great practical and theoretical value. An example is the use of the extremophile, *Thermus aquaticus*, to develop the polymerase chain reaction (PCR) process for the rapid replication of DNA [1]. Studies on organisms living in thermal features may improve our knowledge of the mechanisms of evolution, mineral–microbe relations, biosphere depths, the emergence of life, the origins of ecosystems, and potentially life on other planets [2]. Most work on YNP extremophiles has examined bacteria, archaea, algae, and plants associated with thermal springs, with some limited work on ephydrid flies that feed on algae and cyanobacteria [3,4,5,6,7].

However, these are not the only adapted organisms. We have noted the wetsalts tiger beetle, *Cicindelidia haemorrhagica* (LeConte), walking and feeding in and near thermal springs [8]. There are approximately 2600 species of tiger beetles described. Tiger beetles are cosmopolitan, except for Hawaii, Antarctica, the Maldives, and Tasmania [9,10]. These beetles are found in at least 17 distinctive habitats throughout the United States and Canada. *Cicindelidia haemorrhagica* ranges across the western United States, but is closely associated with water (i.e., lakes, ponds, rivers, sea beaches, tidal flats). Besides Yellowstone [8], *C. haemorrhagica* is also associated with hot springs in California and New Mexico [11,12]. This species is easily recognized by its bright orange abdomen and reduced middle maculation [9,11].

Although *C. haemorrhagica* was first recorded in YNP more than 130 years ago [6], its distribution, ecology, and association with YNP thermal features are not known. Given the harsh environment thermal springs form (with temperatures above 100 °C, varying pH between 1 and 12, iron oxide, hydrogen sulfide, arsenic, etc.) and direct occurrence of beetles in hot thermal pools, the coping mechanisms and adaptations of these beetles [5,13,14] are of interest. Of the few eukaryotic animals associated with YNP thermal features, *C. haemorrhagica* is seemingly the apex invertebrate predator [15]. Outside YNP, *C. haemorrhagica* are not necessarily associated with thermal pools [11], raising the critical question of how YNP *C. haemorrhagica* evolved and the nature of their differences, if any, from populations outside the YNP. Thus, determining the distribution of *C. haemorrhagica,* in association with potentially suitable habitats, thermal and otherwise, within Yellowstone, is an important first step toward examining potential physiological or behavioral differences between populations.

Initial observations of *C. haemorrhagica* within YNP showed the species occurring exclusively in thermal areas, with beetles foraging in heated pools. Given restrictions associated with research in a national park, population assessment was necessary to identify a population from which experimental individuals could be taken. A variety of methods are used to assess tiger beetle populations, (see Person and Vogler for a summary [9]). One option is the use of the mark–recapture technique, in which various calculation approaches are possible [16]. The simplest of these is the Lincoln index, which can provide an accuracy of ±10%, if a sufficient proportion of the population is marked [17]. However, assumptions required by the Lincoln index preclude or complicate its use, particularly the need for a closed population. For tiger beetle species associated with discrete habitats, populations are typically functionally closed. Because *C. haemorrhagica* occurs in YNP in association with thermal springs, its behavior mirrors that of other tiger beetle species with limited spatial distribution in the local population. We explored light attraction as a potential method for trapping *C. haemorrhagica* for marking.

Another option is the use of visual counts, a relative technique used to estimate population. This technique is ideally tied to a more accurate, absolute sampling method, such as mark–recapture [18]. A study by Spomer and Higley [18] used visual counts to estimate abundance on salt-marsh tiger beetles, *Cicindela nevadica lincolniana* Casey. This can be applied when studying *C. nevadica lincolniana* and other insect populations that are highly visible and have strong habitat restrictions. *C. haemorrhagica* meets this requirement by being both limited to saline habitats and having a strong fluvial association. These beetles spend most of their daytime activity within 4 m of the water’s edge on the exposed saline pond and creek banks. Exposed damp areas are needed for finding mates, thermoregulation, and hunting prey. Researchers can, therefore, exploit this behavior by walking in the streams or ponds and counting the majority of beetles present. Consequently, and based on habitat limitations, it seems possible to conduct visual counts for *C. haemorrhagica* in a manner analogous to that used for estimating *C. nevadica lincolniana* populations [18].

In this study, we assessed *C. haemorrhagica* distribution in YNP, examined habitat characteristics and behavior in response to light, and estimated population size at specific locations. This information provides the necessary background information for documenting *C. haemorrhagica’s* exclusive relationship with thermal springs in Yellowstone National Park.

## 2. Materials and Methods

Yellowstone National Park represents a pristine Rocky Mountain environment, including alpine meadows, short grass prairie, subalpine forests, and numerous thermal areas with active hot springs, fumaroles, mudpots, and related volcanic features. The park itself includes a roughly 50 × 70 km caldera, more than 10,000 thermal features, and a mean elevation of 2400 m [14].

### 2.1. Distribution

We conducted all research under YNP research permits #7092 and #8100. To determine the occurrence and distribution of *C. haemorrhagica* and other cicindelid species in YNP, we conducted a presence/absence study of geographically distinct thermal areas. During the summer of 2016, initial observations were made at each thermal feature that was publicly accessible by automobile and foot within YNP. After initial sites containing *C. haemorrhagica* were found, we observed them to determine the time and temperatures of activity, as well as ideal weather conditions. We observed each potential site for 15 min during what we found to be periods of peak beetle activity (09:00–17:00). If there were no beetles active during this time, the site was marked “absent.” However, if weather conditions were not optimal (i.e., heavily overcast, rain, wind, or soil temperatures less than 20 °C), we returned at a different time to confirm the initial observations. If there was beetle activity, the site was marked as “present,” and, conditions permitting, we noted the species, geographic coordinates, temperature measurements of the soil, water, and ambient air, along with altitude, wind current, and humidity. Throughout our field study (2016–2019), we looked for *C. haemorrhagica* occurrence in YNP to determine if they occur in non-thermal areas.

### 2.2. Habitat Characteristics

During the distribution presence/absence study, we observed habitat characteristics, such as soil moisture, presence of prey, predators, and algal and bacterial mats, among others. We recorded soil and water surface temperatures (3 replicates per site) with an Omega HH81A digital thermometer (https://www.omega.com/en-us/, accessed on 3 August 2016) (soil) and FLUKE 62MAX+ infrared thermometer (https://www.fluke.com/en-us, accessed on 3 August 2016) (water). We collected water samples with a 100 mL glass bottle attached to an extendable pole. The pH of the water was tested with an Oakton CON 450 portable meter (https://www.coleparmer.com/, accessed on 3 August 2016) and an AI209 PH20 pH pocket meter (https://aperainst.com/, accessed on 3 August 2016). We conducted temperature and pH tests from three areas within each site; more were conducted if there was a high degree of variation. These observations made it possible to determine ideal habitat and predict the occurrence of beetles in sites throughout YNP. Determining ideal habitats substantially reduced the time spent searching for viable research sites, as YNP contains more than 10,000 geothermal features.

### 2.3. Abundance

Light-trapping and mark–recapture. To assess the abundance of *C. haemorrhagica* in YNP, we initially employed capture procedures following those successfully used in research with *C. nevadica lincolniana* [19]. In the summer of 2017, we placed light traps for *C. haemorrhagica* at Angel Terrace in YNP using both a 15 W black light (UV) and 175 W mercury vapor bulbs. We chose three nights when nighttime air temperatures exceeded 19 °C to ensure beetle activity. Nocturnal activity for tiger beetle adults is typically limited to air temperatures above 15.5 °C; however, given their association with thermal areas, it seems likely that *C. haemorrhagica* could be active at lower ambient temperatures (because of the warming of flight muscles while on heated substrates.)

**Figure 1:**
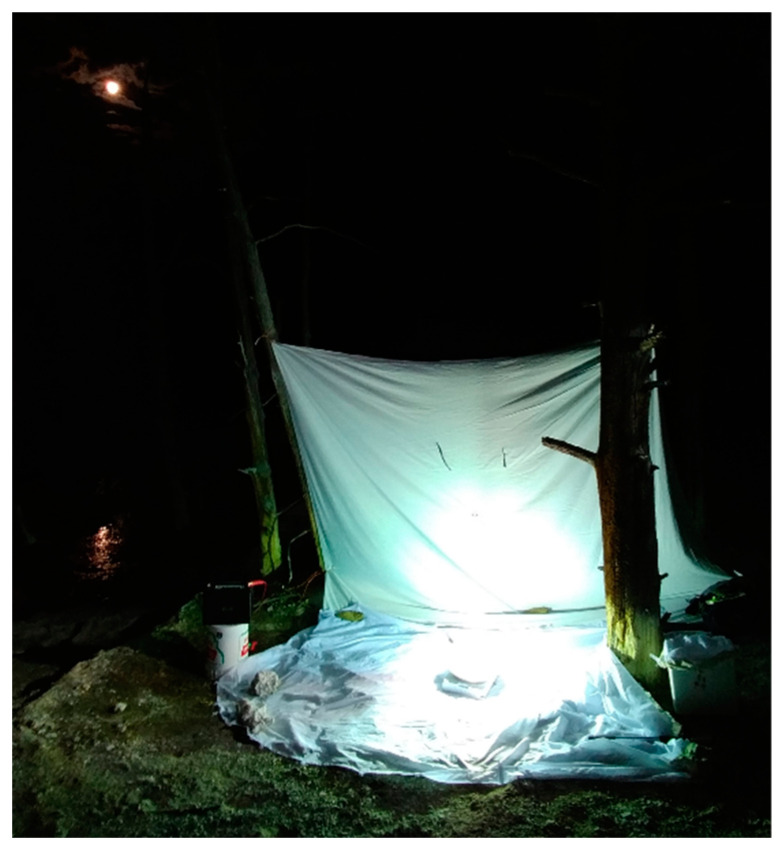
Light-trapping set-up showing portable light and battery, a collection box, and white cloth at a 1 m distance from the thermal spring at Angel Terrace.

We placed the black light and mercury vapor bulb on top of a white cloth sheet that was partially hung between trees and lying flat on the ground, creating an L-shaped set-up (Figure 1). The lights and sheets were placed on the forested ground approximately 5 m from the active thermal pool. The light reflects off the white sheets, therefore increasing its visibility to light-attracted insects. The combination of both UV black light and mercury vapor provides a sufficiently broad spectrum of wavelengths attractive to beetles. Insects landed on the white sheets and were then easily collected.

The collected *C. haemorrhagica* individuals were sexed, and a drop of silver paint was added to the top of the elytra using DecoColor colored pens (https://uchida.com/, accessed on 9 July 2017). The exact location of the droplet differed between beetles and was noted to be able to identify each beetle. In establishing mark–recapture procedures with *C. nevadica lincolniana*, Allgeier [19] found no evidence of increased adult mortality from marking methods (either directly or from increased predation). We placed the marked beetles in a container and released them after marking so they could mix within the population.

Because we had low capture rates the first night, we decided to observe the ratio of marked vs. unmarked beetles with binoculars rather than recapturing them, to gain a better estimate of population size. To double check if *C. haemorrhagica* is indeed minimally responsive to light, we light trapped two more times, on 8 and 10 July 2017.

### 2.4. Phototaxis Experiment

Given the poor capture of *C. haemorrhagica* at black light and observations that the species was poorly attracted to light [11], we performed a phototaxis experiment to determine if *C. haemorrhagica* is attracted to light. We collected *C. haemorrhagica* beetles from four sites within YNP and one site outside of YNP that was not associated with thermal springs. The non-YNP site was at the C.J. Strike Wildlife Management Area in Idaho, which is the closest *C. haemorrhagica* population to those in YNP (566 km between sites). A closely related tiger beetle species, *Cicindelidia punctulata* (Olivier), is known to be attracted to light and was collected from a site in Lincoln, Nebraska, USA. This species was our control to determine if our experimental set-up was correct. Beetles from Angel Terrace, Dragon–Beowulf Hot Springs, and Sizzling Basin were used to represent *C. haemorrhagica* from sites within YNP. The site where *C. punctulata* was collected is located near Memorial Stadium in Lincoln, Nebraska and was labelled “Lincoln”. All experiments were conducted at night (ca. 22:00) with unfed individuals who were tested within 4 days of capture.

In our first experiment, we compared the light attraction of *C. haemorrhagica* from different sites within YNP. Five beetles from Angel Terrace, six beetles from Sizzling Basin and five beetles from Dragon-Beowulf Springs were used. These beetles were collected from YNP, brought back to the lab and kept in an environmental chamber of 29 °C with a 15:9 L/D cycle. This was comparable to the natural cycle in the field, with sunrise around 06:00 and sunset around 21:00.

For the experiment, the beetles were divided by site location and sex, and randomly assigned to a container. For our experiment, we used 473 mL beverage cups that were blacked out with black electrical tape. Each container had two blacked out tubes (25 cm long by 3 cm diameter) inserted on opposite ends. The tubes were covered in black wrapping paper and a pinhead size hole was made on each end. This hole allowed for light penetration in the dark experimental unit. The side that must remain dark was taped off with black electrical tape. The experimental unit was placed into an environmental chamber with a constant temperature of 29 °C to eliminate warmth of the light source as a confounding factor.

By placing the experimental unit in a dark chamber, we eliminated any residual light that might skew our results. After the assigned beetle was placed into the middle cup, we closed the cup and rearing chamber, and allowed the beetle to move around for 10 min. After 10 min, the lid was opened to check if the beetle was found in the middle cup. If so, this was noted along with the sex and site location of the beetle. If the beetle was not found in the middle cup, one tube was clogged with a cotton ball to prevent the beetle from changing location. The set-up was then moved at an angle to move the beetle back into the center and to become visible. If the beetle did not appear, the cotton ball was switched to the other tube, and the same procedure took place. We then noted the sex and site location of the beetle, which tube contained the beetle, and which side contained the light source.

For our second experiment, the experimental set-up remained the same, but it took place the night immediately following the capture of these beetles. We wanted to eliminate any influence that might arise due to our bringing the beetles to the lab and waiting several days before conducting the experiment. There were 3 experimental units per trial, 4 trials total, with 12 beetles at a 50:50 sex ratio. The locations assessed were Idaho, Rabbit Creek, and Dragon–Beowulf Spring.

We analyzed the data from both experiments in SAS ver.9.4 (SAS Institute, Cary, NC, USA) software, with treatments evaluated by *t*-test or Chi-sq as appropriate.

### 2.5. Visual Counts

Visual counts were made of both adults and larval burrows to assess the abundance of *C. haemorrhagica*. Depending on the accessibility and size of the thermal feature, different strategies were used. For the large and relatively accessible sites such as Dragon–Beowulf Springs and Rabbit Creek, we walked side by side and moved along the thermal stream while counting adults. The constant movement and clearly defined sections allowed us to minimize recounts. For less inaccessible and smaller sites, such as Idaho, Sizzling Basin, and Angel Spring, visual counts were made with binoculars, where the area was scanned at a constant motion to minimize recounts as well.

**Figure 2:**
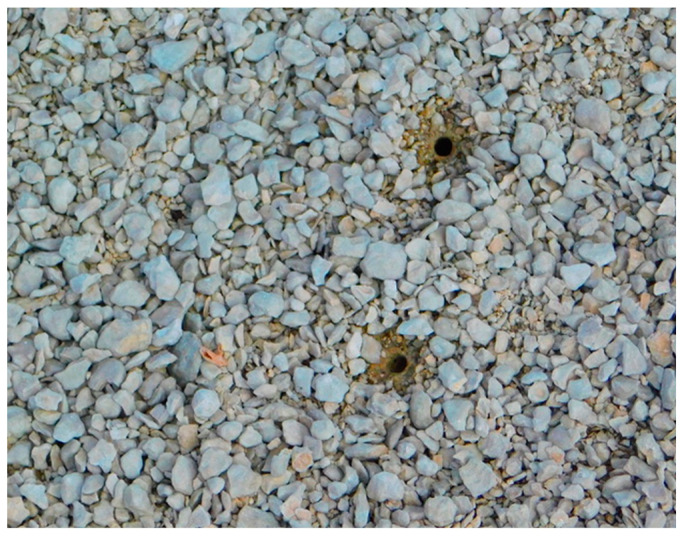
Active burrows of *Cicindelidia haemorrhagica* in Yellowstone National Park. The burrow has a round shape and the area surrounding it is cleared. Photograph by John Bowley at Dragon–Beowulf Springs in 2019.

We noted larval burrows when they had a virtually perfect round-shaped burrow opening with no debris nearby (Figure 2). When burrows are abandoned, they will quickly lose their characteristic shape and debris will build up.

## 3. Results and Discussion

### 3.1. Distribution and Habitat Characteristics

**Figure 3:**
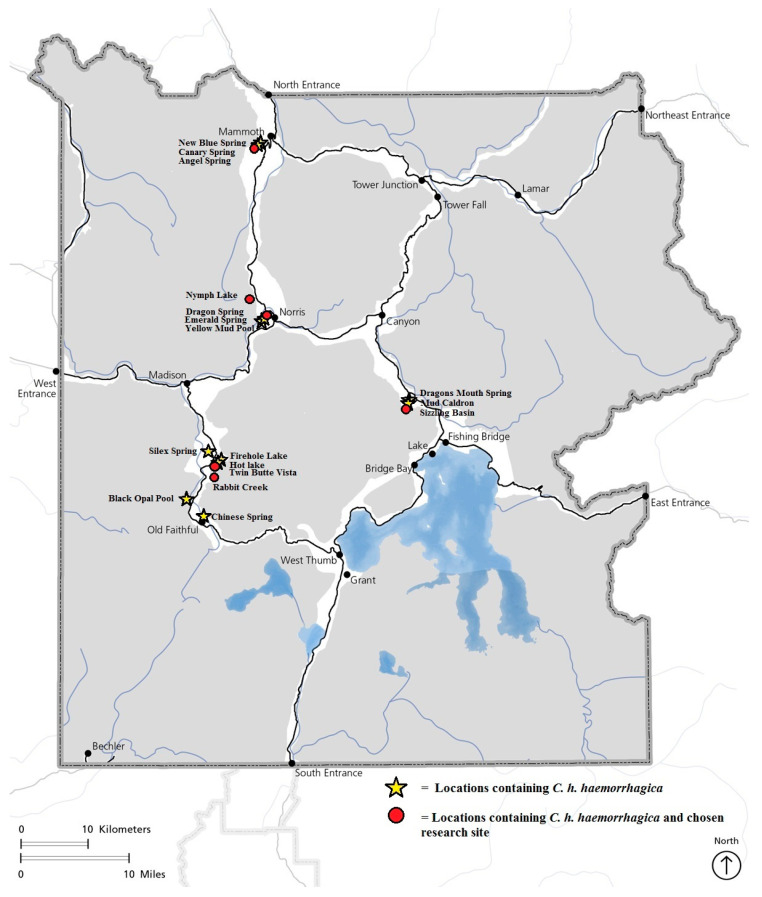
The locations of *Cicindelidia haemorrhagica* within Yellowstone National Park. The yellow stars represent the sites where a thriving *C. haemorrhagica* population was found, the red circles represent the sites containing a thriving population that were chosen as research sites.

After the initial presence/absence study in 2016, *C. haemorrhagica* were found at 10 thermal springs. In surveying for *C. haemorrhagica,* sites were inspected for a minimum of 15 min from the center of the thermal feature to ca. 5 m from the margin of thermal pools. Close focusing binoculars were used to facilitate observations. Throughout our research from 2016 to 2019, we found 7 additional locations containing *C. haemorrhagica* (Table 1, Figure 3) for a total of 17 confirmed locations inside YNP. All thermal springs differed in pH, temperature, chemical composition, and conductivity (Montana State University, 2017). The lowest pH was 2.2 at Sizzling Basin, while the highest pH was 9.5 at Rabbit Creek. The thermal spring associated with *C. haemorrhagica* with the lowest temperature was Yellow Mud Pool (Upper Norris Geyser Basin) at 29.1 °C and the highest was Rabbit Creek Hot Springs (Midway Geyser Basin) at 92.2 °C. Although the source pool of this creek was recorded at 92.2 °C, the beetles were never recorded in temperatures over 70 °C. Conductivity varied from 520 to 4100 µS/cm. We found beetles associated with all three types of thermal springs characterized by calcium carbonate, alkaline silica, and sulfuric acid (Table 1).

These data show that *C. haemorrhagica* can be found in a wide range of habitats. However, the pH, temperature, and conductivity of the spring source do not reflect the actual conditions at which these tiger beetles live. From a number of sites, we noticed beetle activity at slightly less extreme pH and temperature values. In general, beetles occurred in thermal pools at temperatures ranging between 29 °C and 70 °C, and beetles were observed foraging in heated pools in roughly equal numbers to those on the edges of pools.

Not all sites seemingly suitable for *C. haemorrhagica* had beetles. Table 2 indicates locations in which beetles were not found. In no instances were beetles found unless pools of heated water occurred.

We found populations throughout YNP with the exception of northeastern and southern YNP (Figure 3). Yellowstone National Park has an estimated 10,000 thermal features within its boundaries [14]. Many of these thermal features are far from main roads and in rough terrain. We only assessed the thermal features that were easily accessible by automobile or by foot, mainly for the safety of our researchers. It is highly likely that more thermal features will contain *C. haemorrhagica* populations, given the presence of so many thermal features throughout YNP. Northeast YNP currently does not show any sites containing *C. haemorrhagica* either, which is most likely because of a lack of thermal features on that side of the YNP. Interestingly, Mammoth Hot Springs contains *C. haemorrhagica* populations while being an isolated thermal area from the rest of YNP. Dispersal through rivers and lakes seems to be the most likely scenario, although many areas are surrounded by high mountain ranges. The thermal areas in YNP are dynamic and change often due to earthquakes blocking, moving, or creating new thermal features. It is possible that shifts of thermal areas allowed *C. haemorrhagica* to move throughout the YNP.

A common characteristic of nearly all sites was a gradation (a less than five-degree slope) from spring to soil. Sites with a deep and abrupt ridge or dip between soil and water never contained *C. haemorrhagica* populations. We hypothesize that *C. haemorrhagica* needs this thermal gradation and gradual slope into the water for larval habitat and to avoid falling into and succumbing to the heat of the water. The brown, red, and green colorations surrounding thermal springs indicate the presence of various species of bacteria and algae. These algal and bacterial mats often contained ephydrid flies and larvae. The tiger beetles were seen probing through the algal and bacterial mats and actively hunting ephydrid flies. Zach [20] noted that tiger beetles often predate on the ephydrid larvae found in the algal mats. Sites not containing algal or bacterial mats were less likely to contain *C. haemorrhagica*, and adults were often seen feeding on insects that died in the thermal water.

We never observed *C. haemorrhagica* farther than 8 m from a thermal spring. Although there was mention of two different tiger beetle species, *C. oregona* near thermal springs [20] and *C. decemnotata* near the Gardiner River (Ostovar, unpublished), we were unable to confirm this. Regardless, *C. haemorrhagica* seems to be the most abundant tiger beetle species and apex invertebrate predator in these areas of YNP. It feeds in algal and microbial mats, and feeds on invertebrate larvae (e.g., ephydrids, and chironomids) and invertebrate adults (i.e., ephydrids, ants, other flies, other beetles, wolf spiders). The success of *C. haemorrhagica* could be due to the additional heat offered in the winter by the thermal springs to overwintering larvae and adults. We noticed beetles oviposit close to the thermal features (<5 m), which was confirmed by the presence of different sizes of larval burrows within that same distance. We found no evidence of burrows farther than 5 m from thermal pools, and all burrows occurred in thermal soils (i.e., soils with subsurface heating, and a surface temperature range of 24.5 °C to 42.3 °C). It is possible that the heat of the thermal soil and water reduces mortality in the winter compared with larval burrows that are not associated with thermal springs. In addition, many insects succumb to the steam or heat of the thermal water and float toward the edges, where adult tiger beetles will forage [15]. This additional source of food may also explain the seemingly exclusive association with thermal springs. Having determined where *C. haemorrhagica* is likely to occur within the YNP, we wanted to determine how to best estimate abundance. Abundance information was necessary for determining where individuals could be sampled for bioassays (not reported here) without deleteriously impacting populations.

### 3.2. Abundance Estimations

Mark–recapture. Insect population estimates are commonly associated with detection (presence/absence) for quarantine, with determining density for pest management decision-making or ecological research, or with density monitoring of threatened or endangered species [16]. Sampling methods must be non-destructive because of small population sizes, and the range of sampling techniques may be limited because of habitat, and low total population sizes (e.g., 1000–10,000 individuals). Most insect population estimates profit from the statistical law of large numbers (provided sampling is sufficient) given the large sizes of most insect populations. In contrast, insects associated with highly specific habitats (like most extremophiles) have small populations and will have greater uncertainty in population estimates.

On 6 July 2017, four *C. haemorrhagica* were collected at Angel Terrace using both black light and mercury vapor bulbs. Sunset occurred at 21:12 and lights were set out at 21:33. The first beetle was caught at 22:49 and the last beetle at 22:57. The ambient air temperature was 20.7 °C at the start of the light-trapping, and slowly decreased to 18.5 °C at the end of trapping at 00:00. The beetles were marked with silver paint and released at the end of light trapping. On 7 July 2017, the beetles were observed with binoculars to count how many were unmarked and how many marked beetles were present in the population. We found 39 unmarked and one marked beetle. Following the simple Lincoln index:

$$N=\frac{an}{r}$$

where *N* = the estimated number of individuals within the population, *a* = the total number of marked individuals in the first sample, *n* = the total number of individuals collected in the second sample, and *r* = the total number of recaptured individuals [16]. When we applied this Lincoln index to our mark–recapture efforts we found:

$$N=\frac{4\times 40}{1}=160\;\;\;\;\;\;160+3=163$$

We marked 4 beetles and observed 40 in the field, of which only 1 contained the silver paint marking. We calculated at least 160 individuals in our population, and if we include the 3 previously marked ones, we arrive at a population size of at least 163 individuals at Angel Terrace. Due to the difference in capture method (light trapping versus observation) and due to the highly specific habitat, there is some uncertainty in the accuracy of this population estimation.

On 8 July 2017, two *C. haemorrhagica* beetles were caught at Angel Terrace using blacklight and mercury vapor. The sun set at 21:11 and the lights were set out at 21:51 with an ambient air temperature of 20 °C. The temperature slowly decreased to 18.1 °C at the ending time of 00:00. These beetles were marked with yellow paint and released. No previously marked beetles were recaptured. On 10 July 2017, one *C. haemorrhagica* beetle was caught at Angel Terrace using only blacklight. Ambient air temperatures were not ideal as they ranged from 14.7 to 15.4 °C, below optimal foraging temperature for tiger beetles. This beetle was not previously marked and was given an orange marking. Later visual observations showed no marked beetles in the population, confirming that there is a larger population at Angel Terrace than what is visible to us when conducting a visual count.

### 3.3. Phototaxis Experiment

To confirm if *C. haemorrhagica* adults are unresponsive to light, we tested the attraction to light of individuals from YNP, outside YNP (Idaho), and of *C. punctulata* in 2017, 2018, and 2019. Beetles were collected from six different sites with an approximate 50:50 sex ratio. We tested 13 *C. haemorrhagica* beetles (7 males, 6 females) from Angel Terrace, of which 6 were attracted to the light, 6 avoided the light, and 1 did not make a choice. Of the 25 *C. haemorrhagica* beetles (12 males, 13 females) from Dragon–Beowulf Springs, 13 were attracted to light, 4 avoided the light, and 8 did not make a choice. We tested 12 *C. haemorrhagica* beetles (6 males, 6 females) from Rabbit Creek, of which 2 were attracted to the light, 2 avoided the light, and 8 did not make a choice. For individuals from Sizzling Basin, we tested 6 *C. haemorrhagica* (3 males, 3 females), of which 1 beetle went to the light side, 3 went to the dark side, and 2 did not make a choice. Of the 12 *C. haemorrhagica* (8 males, 4 females) tested from Idaho, which are not associated with thermal springs, 3 were attracted to the light, 2 avoided the light, and 7 did not make a choice. The light-attracted species, *C. punctulata*, was collected in Lincoln, Nebraska and 13 beetles (7 males, 6 females) were used in the light experiment and, of these, 9 were found in the tube with light, 1 was found in the darkened tube, and 3 beetles did not choose and remained in the middle cup.

**Figure 4:**
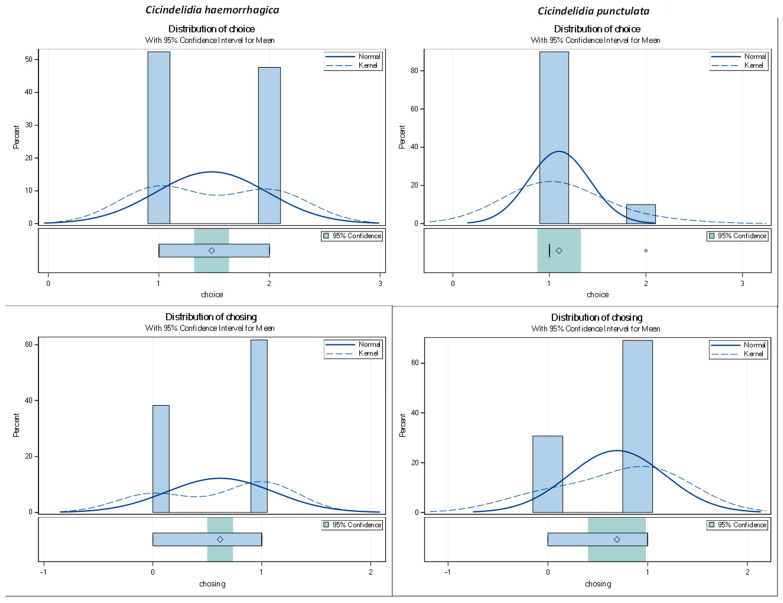
Phototaxis results showing in which tube the beetle was located after 10 min (0 = beetle did not choose the lightened tube, 1 = beetle was found in lightened tube, 2 = beetle was found in darkened tube.) A = Light or dark choice for *C. haemorrhagica*, B = Light or dark choice for *Cicindelidia punctulata*, C = Light or not light (dark or no choice) for *Cicindelidia haemorrhagica*, D = Light or not light (dark or no choice) for *C. punctulata*.

We saw no significant difference in light or dark preference between sexes (ChiSq, *p* = 0.24) or between years (ChiSq, *p* = 0.73). *Cicindelidia punctulata* was significantly attracted to light, as expected. When a choice was made, approximately 90% of *C. punctulata* were found in the tube with the light source, while approximately 10% were found in the dark tube (*t*-test, *p* < 0.0001) (Figure 4). When comparing light preferences versus dark or no preference, 69% were indifferent to light while 31% were attracted to light (*t*-test, *p* < 0.0002). Surprisingly, the light or dark preference was significant for *C. haemorrhagica*. For *C. haemorrhagica*, approximately 52% of the choices were made toward the darkness, while approximately 48% were made toward the light (*t*-test, *p* < 0.0001). When including the no choice scenario where beetles remained in the middle cup, about 68% appeared indifferent, whereas 32% chose the lighted side (*t*-test, *p* < 0.0001). These results support our hypothesis that *C. haemorrhagica* show inconsistent and limited attraction to light.

Certain insects use the transverse orientation to navigate at night. They can fly straight by keeping a strong light source (usually the moon) at a constant angular relationship [21]. Other insects will use the reflection from the moon off the water to locate new water sources. Perhaps, within YNP, it is too dangerous to locate new water sources, as thermal springs can be deadly. If navigating at night and locating new water sources is detrimental to survival, selection may have occurred for those that did not navigate at night. Pearson et al. [11] have noted that *C. haemorrhagica* are occasionally, but not regularly, attracted to lights. Therefore, the reduced attraction to light does not seem to be a consequence of life in YNP. In addition, Pearson and Lederhouse [12] have noted the inactivity of *C. haemorrhagica* when temperatures were below 21.2 °C. Nighttime temperatures in YNP rarely exceed this threshold and this could be another explanation for the beetles’ inactivity and minimal-to-no light attraction at night.

### 3.4. Census and Observation

These limitations (failure of light trapping and inability to adult sample in the delicate thermal areas where beetles occurred) left us with observation and census as the only remaining techniques. Table 3 presents data from three census types: Moving counts of all observable adults, moving counts of all observable active larval burrows, and timed counts of adults made from a single location. Table 4 provides calculated estimates of population size in 2017 using ratios calculated in 2019. Because observational data like these always underestimate population size, these estimates are conservative—meaning the actual population sizes will be larger. Given the crudeness of the technique, the best use of these values is most likely as an index to the order of magnitude of the population size, rather than as a specific number. Thus, we interpret these results as indicating that *C. haemorrhagica* populations at most specific springs likely ranged between 500–1500 individuals. We estimated a population size of 676 individuals for Dragon–Beowulf Spring, 538 individuals for Rabbit Creek, 676 individuals for Angel Terrace, 423 individuals for Sizzling Basin, and 1048 individuals in Idaho. Larger areas containing multiple springs (such as the Norris Geyser Basin) probably have populations of many thousands of individuals.

## 4. Conclusions

In summary, *C. haemorrhagica* in YNP were exclusively associated with thermal areas, in at least 17 sites that were readily accessible by car and foot. Populations at measured locations were in excess of 500 individuals, and individuals were observed within 8 m of thermal springs. Beetles occurred in alkaline and acid springs, and were noted at temperatures as high as 70 °C. How *C. haemorrhagica* manages to survive under such harsh conditions is the most compelling question that emerges from this study.

## Figures and Tables

**Table 1:** Locations and habitat characteristics of sites containing *Cicindelidia haemorrhagica* within Yellowstone National Park (Figure 3).

Name	Latitude	Longitude	pH *	Temperature (°C) *	Conductivity (µS/cm) *	Year of Beetle Location
New Blue Spring	44.96909	−110.70667	5.83	42	2500	2016
Canary Spring	44.96742	−110.70532	6.84	61.1	2192	2016
Yellow Mud Pool	44.72049	−110.70629	2.47	29.1	2300	2016
Emerald Spring	44.72565	−110.7042	4.55	83.3	2600	2018
Mud Caldron	44.62394	−110.4333	2.7	66.6	3300	2016
Sizzling Basin	44.62181	−110.4339	2.2	37.3	4100	2016
			2.73 ± 0.18 **	41.8 ± 0.5 **		
Dragons Mouth Spring	44.62516	−110.4349	5.4	68.5	618	2016
Black Opal Pool	44.48508	−110.85352	9.03	30.5	1800	2016
Firehole Spring	44.5351	−110.802	8.2	83.6	1800	2016
Hot Lake	44.54384	−110.7886	8.21	62.6	520	2016
Silex Spring	44.55019	−110.80585	8.44	79.3	45	2016
Chinese Spring	44.4618	−110.829167	N.A.	N.A.	N.A.	2019
Angel Terrace	44.96554	−110.70987	7.38	61	2600	2017
			7.13 ± 0.15 **	44.92 ± 11.48 **		
Dragon–Beowulf Springs	44.73182	−110.71092	3.22	66.5	3500	2017
			2.88 ± 0.17 **	49.31 ± 4.42 **		
Twin Butte Vista	44.53553	−110.7972	8.59	87.9	1600	2017
			8.95 ± 0.13 **	52 ± 10.17 **		
Rabbit Creek	44.52097	−110.8125	9.5	92.22	2500	2019
			9.35 ± 0.17 **			
Nymph Lake	44.75178	−110.72268	2.49	71	1800	2017
			2.82 ± 0.03 **	52.67 ± 6.09 **		

* Values of thermal features taken from the Yellowstone National Park Research Coordination Network at http://www.rcn.montana.edu (9 July 2019). N.A. = values not available. ** Own collected values of thermal features where beetles were seen.

**Table 2:** Locations of sites without *Cicindelidia haemorrhagica* within Yellowstone National Park inspected for a minimum of 15 min per site in late July 2016.

Undine Falls	Norris Geyser Basin	Midway Geyser Basin
Wraith Falls	Steamboat Geyser	Grand Prismatic Spring
Blacktail Plateau Drive	Cistern Spring	Excelsior Geyser Basin
Petrified Tree	Black Pit Spring	Opal Pool
Tower Fall	Echinus Geyser	Turquoise Pool
Washburn Hot Springs Overlook	Arch Steam Vent	Firehole River
Cayon Village Upper and Lower Falls	Mystic Spring	Roaring Mountain
Fountain Flat Drive	Porkchop Geyser	Beaver Lake
Firehole Canyon Drive	Vixen Geyser	Indian Creek
Artists Paintpots	Palpitator Spring	Sheepeater Cliff
Old Faithful	Corporal Geyser	**Mammoth Hot Spring Area**
**Fountain Paint Drive**	Veteran Geyser	Palette Spring
Black Warrior Lake	Fearless Geyser	Cupid Spring
Pink Cone Geyser	Monarch Geyser	Cleopatra Terrace
White Dome Geyser	Minute Geyser	Orange Spring Mound
Firehole Spring	Congress pool	**Mud Volcano Area**
Surprise Pool	Porcelain Springs	Mud Caldron
**Fountain Paint Walk**	Sunday Geyser	Mud Geyser
Spasm Geyser	Constant Geyser	Cooking Hillside
Clepsydra Geyser	Whirligig Geyser	Churning Caldron
Morning Geyser	Pinwheel Geyser	Sour Lake
Fountain Geyser	Whale’s Mouth	Black Dragon’s Caldron
Twig Geyser		Grizzly Fumarole
Red Spouter		
Fumaroles		
Leather Pool		
Fountain Paint Pot		

**Table 3:** Census counts of *Cicindelidia haemorrhagica* from thermal sites in Yellowstone National Park. See methods for specifics on observational methods; spot counts 2018, adult and larval counts 2019.

Location	Moving Adult Count	Moving Larval Count	Spot Adult Count	Total Individuals
Dragon–Beowulf	180	496	40	676
Rabbit	298	240	.	538
Angel	.	.	40	.
Sizzling	.	.	25	.
Idaho	.	.	62	.

**Table 4:** Ratios of adult/larvae, larvae/adult, and spot counts/adult, larva, and total population, based on data in Table 2. For population estimates at Angel, Sizzling, and Idaho 2017, the spot count/total population ratio determined in 2019 was multiplied by measured 2017 spot counts.

	Calculations				
	From Moving Count		From Spot Count		
Location	Adults/Larvae	Larvae/Adults	Adults	Larvae	Total Pop.
Dragon–Beowulf	36%	276%	450%	1240%	1690%
Rabbit	124%	81%			
			**Total population estimates from spot count proportions**		
Angel	.	.	180	496	676
Sizzling	.	.	113	310	423
Idaho	.	.	279	769	1048

## Data Availability

The data presented in this study are available in article.
